# Transient Neonatal Cyanosis Exacerbated by Inhaled Nitric Oxide

**DOI:** 10.1055/a-2821-3305

**Published:** 2026-03-17

**Authors:** Kaquanta Barlow, Charles J. Sadle, Vidhi Jhaveri, Nicole Cacho, Timothy E. Albertson, Payam Vali, Joseph J. Shen, Satyan Lakshminrusimha

**Affiliations:** 1 Department of Pediatrics, UC Davis Children's Hospital, Sacramento, California, United States; 2 Department of Pharmacology and Toxicology, UC Davis Health, Sacramento, California, United States; 3 Department of Internal Medicine, UC Davis Health, Sacramento, California, United States; 4 Department of Emergency Medicine, UC Davis Health, Sacramento, California, United States

**Keywords:** hereditary methemoglobinemia, Hb F-M-Osaka variant, cyanosis, transient neonatal cyanosis, inhaled nitric oxide

## Abstract

**Background:**

Methemoglobinemia in newborns presents with cyanosis and hypoxemia, which can be mistaken for congenital heart disease or pulmonary hypertension.

**Case Report:**

A term infant presented with cyanosis and low SpO _2_ (70s) immediately after birth, despite continuous positive airway pressure (CPAP) and 100% inspired oxygen. The patient was intubated and started on inhaled nitric oxide (iNO) and prostaglandin E1 infusion. Chest X-ray showed bilateral pneumothoraces; the echocardiogram was normal. Arterial blood gases demonstrated normal pH and elevated PaO _2_ . iNO and prostaglandin E1 (PGE1) were discontinued. Attempts to obtain methemoglobin levels via a co-oximeter panel were unsuccessful, presumably due to out-of-range values. The infant's father revealed that he also had transient cyanosis as an infant. The infant was treated with ascorbic acid. A blood sample sent to a reference laboratory a day after discontinuation of inhaled NO showed a methemoglobin level of 10.2%. Targeted gamma globin gene sequencing found a heterozygous likely pathogenic variant in hemoglobin subunit gamma 2 (HBG2) (p.His63Tyr). He was discharged home at 1 week of age on room air.

**Conclusion:**

Hereditary causes of methemoglobinemia should be considered for newborns with persistent cyanosis with low SpO _2_ and elevated PaO _2_ . Detailed family history and avoiding triggers of methemoglobinemia, such as iNO, are the cornerstones of management.

## Introduction

The differential diagnosis of cyanosis with low oxygen saturation by pulse oximetry (SpO _2_ ) in a newborn includes congenital heart disease, respiratory diseases, and persistent pulmonary hypertension of the newborn. We present a case of neonatal cyanosis managed with inhaled nitric oxide (iNO) and prostaglandin E1 (PGE1), where family history obtained from the grandparents was valuable in clinching the diagnosis.

## Case Presentation

A 3.27-kg male infant born at 41 weeks' gestation via spontaneous vaginal delivery in the setting of thick meconium-stained amniotic fluid presented with mild increased work of breathing, cyanosis, and hypoxia immediately after birth. The pregnancy was uncomplicated. Apgar scores were 8 (1 minute) and 8 (5 minutes) due to central cyanosis. The infant had persistent low SpO _2_ with pre-ductal and post-ductal saturations in the low 70s despite CPAP of 6 cm H _2_ O with 100% inspired oxygen. Notably, the initial capillary blood gas (CBG) demonstrated a pH of 7.29 with partial pressure of carbon dioxide (pCO _2_ ) of 24 mm Hg, PO _2_ of 219 mm Hg, and a −15 mEq/L base deficit. Chest X-ray showed a slightly rounded cardiac silhouette and bilateral pneumothoraces. The patient was intubated, and 1 hour later, the CBG showed no improvement (7.3/32/229/17/− 19). He was subsequently started on iNO for suspected pulmonary hypertension. Pre-and post-ductal SpO _2_ worsened to the mid 60s. There was concern for congenital heart disease, so the decision was made to start PGE1 and to transport to a Level IV neonatal intensive care unit (NICU) for further evaluation and management.

**Fig. 1:**
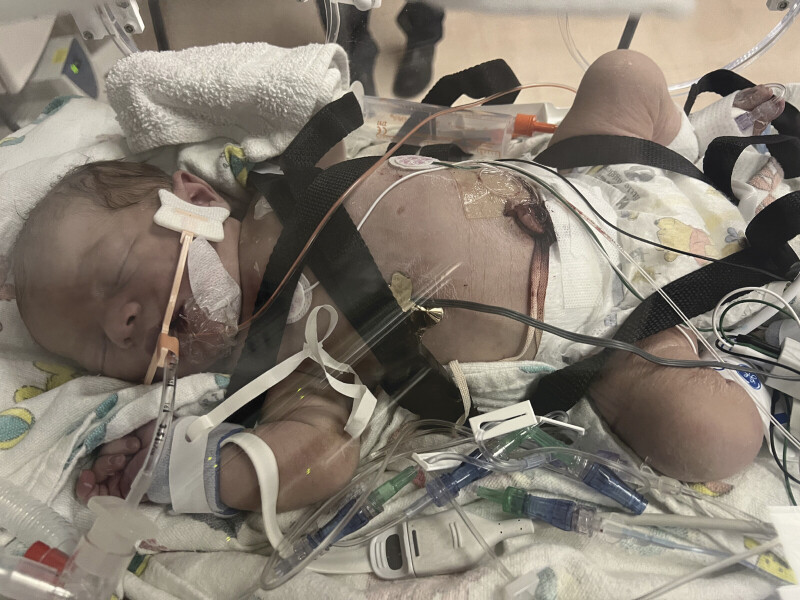
Infant at time of transfer to tertiary care center demonstrating significant cyanosis.

On admission to the NICU, pre- and post-ductal saturations ranged from 67% to 72% on 100% O _2_ . The infant presented with shock and required multiple fluid boluses, norepinephrine, and hydrocortisone ( Fig. 1 ). A stat echocardiogram demonstrated normal cardiac activity and structure without signs of pulmonary hypertension. An arterial blood gas was obtained and continued to show a normal pH with an elevated PaO _2_ (302 mm Hg). Within 2 hours of arrival, iNO and PGE1 were discontinued. Serum electrolytes, liver function tests, complete blood count, reticulocyte studies, bilirubin, and coagulation profile were within limits. Additionally, attempts to obtain methemoglobin levels via the blood gas lab co-oximeter panel were unsuccessful due to out-of-range values.

The father and paternal grandmother disclosed that the father was cyanotic at birth, and that the cyanosis resolved around 3 months of age. The infant's paternal grandmother recalled that the medical team considered administering methylene blue to her son but ultimately elected not to proceed. There was no known history of other hemoglobinopathies, including glucose-6-phosphate dehydrogenase (G6PD) deficiency. Toxicology, Hematology, and Genetics were consulted. The decision was made to administer ascorbic acid and to consider methylene blue if the infant's condition worsened. The toxicology team recommended avoiding oxidizing agents, including but not limited to lidocaine, nitrites, sulfonamide antibiotics, aniline dyes, and dapsone. 1 The genetics team recommended targeted gamma globin gene sequencing, which returned with a heterozygous likely pathogenic variant in HBG2 (p.His63Tyr).

The infant was weaned off norepinephrine once blood pressure stabilized 10 hours after arrival. It was around this time that the clinical skin exam improved from “pale, with gray dusky undertone” to a “general cyanosis.” He was extubated 1 day after arrival, and FiO _2_ was weaned to maintain normal PaO _2_ and SaO _2_ regardless of SpO _2_ in the mid 60s to 70s. A methemoglobin B level was sent to an outside lab; it was obtained a day after discontinuation of iNO, and returned elevated at 10.2, sulfhemoglobin B level 0, and G6PD level was normal. He was weaned to room air on day of life 3 without any increased work of breathing with baseline pre- and post-ductal saturations around 67% to 75%. Hydrocortisone was discontinued on day of life 4. He was discharged home at 1 week of age on full oral feeds. SpO _2_ on the day of discharge ranged between 54% and 74%, and he continued to have “central cyanosis.” Outpatient follow-up was scheduled with genetics and hematology. The parents reported decreased cyanosis at 1 month of age, with complete resolution by 3 months of life. Providers at well newborn appointments did not comment on the infant's cyanosis or reassess PaO _2_ .

## Discussion

The differential diagnosis of cyanosis with a wide discrepancy between SpO _2_ and PaO _2_ includes methemoglobinemia. The approach to diagnosis began by discerning acquired versus inherited methemoglobinemia, followed by a careful review of clinical and family history.

Methemoglobinemia results from impaired reduction of ferric (Fe ^3+^ ) iron to ferrous (Fe ^2+^ ) iron within hemoglobin, most commonly due to cytochrome b _5_ reductase deficiency (CYB5R3). Methemoglobin is unable to bind oxygen and also increases the oxygen affinity of remaining normal hemoglobin, shifting the oxyhemoglobin dissociation curve to the left and impairing oxygen release to tissues. As a result, arterial PaO _2_ may be normal or elevated, while functional oxygen saturation and oxygen delivery are reduced, producing persistent cyanosis and apparent hypoxemia. Nitric oxide binds to oxyhemoglobin and is rapidly converted to nitrite and nitrate, a reaction that oxidizes the heme iron from the Fe ^2+^ to the ferric Fe ^3+^ state, thereby increasing methemoglobin formation.

Treatment aims to enhance the reduction of methemoglobin back to functional hemoglobin. Ascorbic acid acts as a non-enzymatic reducing agent, providing a slow but sustained pathway for methemoglobin reduction, and is often used for chronic management. Methylene blue serves as an artificial electron carrier, accelerating NADPH-dependent reduction of methemoglobin via the hexose monophosphate pathway; it is reserved for symptomatic or severe cases and requires intact G6PD activity. Methylene blue can result in hemolysis in patients with G6PD deficiency. 1 In shared decision-making with the family, due to the relative G6PD state of neonates, the family decided on ascorbic acid treatment, which is a slower method to reverse methemoglobinemia.

The role of iNO-induced exacerbation of hereditary methemoglobinemia was entertained in this patient. In patients without hereditary methemoglobinemia, iNO typically causes a mild elevation in methemoglobin. A 2010 study of 163 infants treated with iNO found 10% ( *n* = 16) of the babies to have a methemoglobin level of 2.5% to 5%, and only one child with a methemoglobin level >5%. 2 Deterioration in SpO _2_ after initiating iNO can be due to left ventricular dysfunction, congenital heart disease such as total anomalous pulmonary venous return, and rarely congenital methemoglobinemia. Our patient's methemoglobin level of 10.2% was greater than the levels seen in acquired methemoglobinemia from treatment with iNO.

**Fig. 2:**
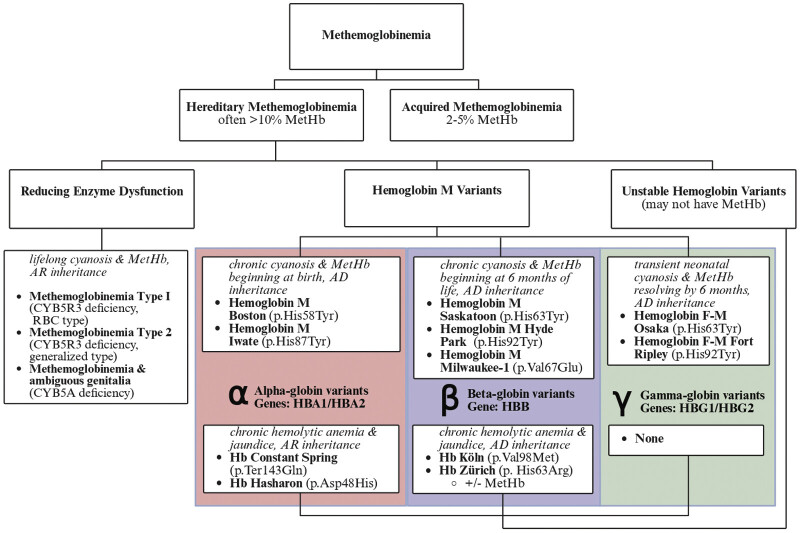
Diagnostic algorithm of methemoglobinemias, including common representative hereditary methemoglobinemia variants.

With acquired methemoglobinemia unlikely, hereditary methemoglobinemias were considered. There is nomenclature confusion about “congenital methemoglobinemia” because the term refers to a specific subset of hereditary methemoglobinemia caused by CYB5R3 enzyme deficiency. Hereditary methemoglobinemia provides a more appropriate broad term for all genetic variants that cause methemoglobinemia. Hereditary methemoglobinemias can be first divided into enzymatic variant causes and hemoglobin variant causes ( Fig. 2 ). The most common cause of hereditary methemoglobinemia is due to enzyme variants in CYB5R3, which cause the autosomal recessive conditions: Congenital Methemoglobinemia Types 1 and 2. Hemoglobin naturally oxidizes into methemoglobin, which cannot bind oxygen, and the CYB5R3 enzyme is the primary physiological pathway that reduces this methemoglobin back into functioning hemoglobin. Type 1 Congenital Methemoglobinemia (RBC-type) is caused by CYB5R3 variants that result in loss of function of the erythrocyte-only CYB5R3 enzyme, resulting in mild persistent methemoglobinemia throughout life, which accounts for 90% of disease-causing variants. Congenital Methemoglobinemia Type 2 is caused by CYB5R3 variants that result in diffuse loss of CYB5R3 enzyme, which leads to a more severe multisystemic phenotype of microcephaly, spasticity, polycythemia, and significant developmental delays. Our patient's presentation was inconsistent with a reducing enzyme cause of methemoglobinemia for several reasons: (1) The methemoglobin level was 10.2% obtained during the neonate's second day and CYB5R3-related methemoglobinemia patients have levels between 20% and 30%, 3 (2) the family history was consistent with autosomal dominant inheritance rather than the autosomal recessive inheritance seen in CYB5R3, and (3) the father was unaffected in adulthood, lacking any exertional dyspnea, headaches, or fatigue, which are commonly seen in adults with CYB5R3-related methemoglobinemia.

Hereditary methemoglobinemia can also be caused by hemoglobin variants, which can be further subdivided into “hemoglobin M variants” and “unstable hemoglobin variants” ( Fig. 2 ). Hemoglobin M variants (HbM variants) are caused by point mutations in the globin gene that specifically disrupt the structure of the iron-containing heme pocket, which predispose autoxidation/methemoglobin formation. Unstable hemoglobin variants differ from HbM variants because unstable hemoglobin variants occur outside of the heme pocket and destabilize hemoglobin formation, resulting in hemolytic anemia and jaundice, with methemoglobinemia. 3 Our infant lacked hemolytic anemia or jaundice, further narrowing the differential to hemoglobin pocket variants (HbM variants).

Hemoglobin F is composed of four heme groups—two α (alpha) subunits and two γ (gamma) subunits, whereas hemoglobin A is composed of two α and two β (beta) subunits. The genes responsible for the four proteins that comprise fetal hemoglobin are *HBA1* , *HBA2* , *HBG1* , and *HBG2* .

Hereditary methemoglobinemia caused by disruption of the heme pocket (HbM variants) has distinct presentations depending on whether α, β, or γ-globin is affected ( Fig. 2 ). Because α-globin comprises 50% of adult hemoglobin, hereditary methemoglobinemia caused by α-globin variants has symptoms persisting into adulthood, which the proband's father did not report. Because β-globin is absent from fetal hemoglobin and comprises 50% of adult hemoglobin, hereditary methemoglobinemia caused by β-globin variants presents with symptom onset after 6 months of age, which is inconsistent with our patient's symptom onset at birth. Because γ-hemoglobin comprises 50% of fetal hemoglobin and is absent from adult hemoglobin, hereditary methemoglobinemia caused by γ-globin variants (HBG1 and HBG2) results in cyanosis at birth, which completely resolves before 6 months of life, when a person's hemoglobin has fully transitioned from the fetal (α–γ) hemoglobin to the adult (α–β) hemoglobin.

Due to the father's history of transient neonatal cyanosis with possible methemoglobinemia, there was specific clinical suspicion for an autosomal dominant variant in a γ-globulin subunit. Suspicion prompted targeted γ-globin (HBG1 and HBG2) gene sequencing, which returned with a likely pathogenic heterozygous variant in HBG2 (p.His63Tyr). HBG2 (p.His63Tyr) variants, commonly known as Hb F-M Osaka variant, are reported to cause transient neonatal cyanosis (MIM# 613977). 4 5 6

## Conclusion

Hereditary causes of methemoglobinemia should be considered in the differential diagnosis for newborns with persistent cyanosis and hypoxemia in the setting of normal laboratory values and imaging.

Neonatal methemoglobinemia presents with cyanosis and low SpO _2_ and can be mistaken for congenital heart disease or hypoxemic respiratory failure. Though the majority of methemoglobinemia is due to an acquired process such as medication side effects, hereditary methemoglobinemias cause distinct clinical presentations that, if identified, can alter acute and chronic management of the cyanotic infant. Methemoglobinemia secondary to hemoglobinopathies involving the γ-chain is transient during early infancy and does not recur beyond late infancy. iNO, an FDA-approved pulmonary vasodilator, can worsen methemoglobinemia and exacerbate hypoxemia in the presence of hereditary methemoglobinemia.

Careful clinical and family history (including a discussion with grandparents) can help discern whether the hereditary methemoglobinemia is caused by reducing enzyme variants, heme pocket variants, or unstable hemoglobin variants, resulting in appropriate genetic testing, earlier diagnosis, and improved care outcomes.
